# The Impact of Excessive Body Weight and Foot Pronation on Running Kinetics: A Cross-Sectional Study

**DOI:** 10.1186/s40798-023-00663-8

**Published:** 2023-12-06

**Authors:** Amir Ali Jafarnezhadgero, Azin Jahangirpour, Hamed Parsa, Heidar Sajedi, Urs Granacher, Anderson Souza Oliveira

**Affiliations:** 1 Department of Sport Biomechanics, Faculty of Educational Sciences and Psychology, University of Mohaghegh Ardabili, Ardabil, Iran; 2 Department of Physical Education and Sports Science, İstanbul Esenyurt University, Istanbul, Turkey; 3 Department of Sport and Sport Science, Exercise and Human Movement Science, University of Freiburg, Sandfangweg 4, 79102 Freiburg, Germany; 4 Department of Materials and Production, Aalborg University, Fibigestræde 16, 9220 Aalborg, Denmark

**Keywords:** Free moment, Gait analysis, Ground reaction forces, Loading rate, Obesity

## Abstract

**Background:**

Running exercise is an effective means to enhance cardiorespiratory fitness and body composition. Besides these health benefits, running is also associated with musculoskeletal injuries that can be more prevalent in individuals with excessive body weight. Little is known regarding the specific effects of overweight and foot pronation on ground reaction force distribution during running. Therefore, this study aimed to investigate the effects of overweight/obesity and foot pronation on running kinetics.

**Methods:**

Eighty-four young adults were allocated to four experimental groups: non-excessive body weight/non-pronated feet; non-excessive body weight/pronated feet; overweight or obesity/ non-pronated feet and overweight or obesity/pronated feet. Biomechanical testing included participants to run at ~ 3.2 m/s over an 18-m walkway with an embedded force plate at its midpoint. Three-dimensional ground reaction forces were recorded and normalized to body mass to evaluate running kinetics from 20 running trials. Test–re-test reliability for running speed data demonstrated ICC > 0.94 for each group and in total.

**Results:**

The results indicated significantly lower vertical impact peak forces (*p* = 0.001, effect size = 0.12), shorter time to reach the vertical impact peak (*p* = 0.006, effect size = 0.08) and reduced vertical loading rate (*p* = 0.0007, effect size = 0.13) in individuals with excessive body weight (overweight or obesity/non-pronated feet group and overweight or obesity/pronated feet) compared with individuals non-excessive body weight (non-excessive body weight/non-pronated feet and non-excessive body weight/pronated feet). Moreover, the excessive body weight groups presented lower peak braking (*p* = 0.01, effect size = 0.06) and propulsion forces (*p* = 0.003, effect size = 0.09), lower medio-lateral loading rate (*p* = 0.0009, effect size = 0.12), and greater free moments (*p* = 0.01, effect size = 0.07) when compared to the non-overweight groups. Moreover, a significant body mass by foot pronation interaction was found for peak medio-lateral loading rate. Non-excessive body weight/pronated feet, excessive body weight/non-pronated feet and excessive body weight/pronation groups presented lower medio-lateral loading rates compared to non-excessive body weight/non-pronated feet (*p* = 0.0001, effect size = 0.13).

**Conclusions:**

Our results suggest that excessive body weight has an impact on ground reaction forces during running. We particularly noted an increase in medio-lateral and torsional forces during the stance phase. Individuals with excessive body weight appear to adapt their running patterns in an effort to attenuate early vertical impact loading.

**Supplementary Information:**

The online version contains supplementary material available at 10.1186/s40798-023-00663-8.

## Key Points

1. The results indicated lower vertical impact peak forces, shorter time to reach the vertical impact peak and reduced vertical loading rate in individuals with excessive body weight compared with non-excessive body weight individuals.
2. A significant body weight by foot pronation interaction was found for peak medio-lateral loading rate.
3. Pronation groups presented greater impulses when compared to the non-pronation groups.

**Supplementary Information:**

The online version contains supplementary material available at 10.1186/s40798-023-00663-8.

## Background

Running is an effective exercise for body mass management to fight overweight (e.g., body mass index [BMI] between 25 and 29.9) or obesity (BMI ≥ 30) [1]. However, the repetitive mechanical loading from running may impose a greater risk to sustain acute and/or overuse injuries, particularly in overweight and obese individuals [2]. Moreover, there is evidence that obese compared with non-obese military recruits develop more musculoskeletal injuries following high volumes of speed marching and running [3]. Some factors related to a higher running-related injury incidence are a sudden increase in training volume, excessive body weight and/or poor running technique [4]. Since overweight and/or obesity constitute an additional injury risk factor for recreational runners, it is relevant to understand the influence of excessive body weight on running mechanics, to elucidate protective factors that may help minimize injury risks.

Studies comparing running mechanics from non-excessive body weight and individuals with excessive body weight are scarce [5, 6]. With the term excessive body weight, we refer to overweight (25 ≤ BMI < 30) and obese (BMI ≥ 30) individuals. It has previously been shown that overweight influences running motion patterns, leading to greater peak knee flexion/extension moments in children [5], as well as increased peak hip abduction moments in children [5] and adults [6]. Moreover, adults with excessive body weight also present increased peak hip flexion/extension moments [6]. Such kinetic characteristics may be influenced by reduced center-of-mass excursions and increased vertical stiffness in obese runners [6]. Moreover, increased stride width and longer stance times have been noted in individuals with excessive body weight compared with non-excessive body weight peers [6]. Furthermore, excessive body weight leads to reduced normalized (to body mass) peak ground reaction forces (GRFs) and vertical loading rates (LR) and absolute impulses [6]. Conversely, young overweight or obese individuals present increased absolute vertical LR and absolute peak vertical forces when compared to their peers with non-excessive body weight [5]. Therefore, it appears timely and imperative to gather knowledge on running mechanics in individuals with excessive body weight since their musculoskeletal system manages greater absolute forces.

In addition to excessive body weight, segment lower limb alignment might constitute another relevant risk factor for musculoskeletal running injuries [7]. Excessive foot pronation is one type of segment malalignment, in which the foot rolls inward during the walking or running stance phase, increasing medial forces in the midfoot during walking and lateral forces in the forefoot during both walking and running when compared to individuals with non-pronated foot alignment [8]. In addition, foot pronation contributes to lower extremity misalignment due to excessive tibial and hip internal rotation during walking and running [9, 10], potentially increasing the risk of sustaining musculoskeletal injuries [11]. The incidence of foot pronation in overweight/obese individuals is significant [12, 13], leading to the medial tibial stress syndrome, patellofemoral pain, and low back pain during running [10]. However, it is challenging to estimate the influence of overweight or foot pronation on running biomechanics because both conditions may occur simultaneously in runners. Therefore, isolating overweight and foot pronation as single factors allows to deepen our understanding of running kinetics.

The aim of this study was to investigate the effects of excessive body weight and foot pronation on running kinetics. The main hypothesis is that individuals with both excessive body weight and pronated feet would produce higher loading characteristics (GRFs, vertical LR, and free moment [FM]) during running than individuals with non-excessive body weight and those with non-pronated feet [5].

## Methods

### Participants

Eighty-four sedentary young adults were allocated into four experimental groups. NN: Individuals with non-excessive body weight (e.g., 20 ≤ BMI < 25 kg/m^2^) and non-pronated feet (e. g., 5 < navicular drop < 10 mm, foot posture index between 0 and 6); NP: individuals with non-excessive body weight and pronated feet (e.g., 19 > navicular drop > 10 mm, 12 ≥ foot posture index > 10); ON: Individuals with excessive body weight (e.g., 35 ≥ BMI ≥ 25 kg/m^2^) and with non-pronated feet; OP: Individuals with excessive body weight and foot pronation. In the current study, a modified version of the navicular drop described by Brody was used to determine the sagittal plane displacement of the navicular between seated position and standing on one leg [14]. During testing, participants were seated on a chair with both feet flat on the ground and knees flexed at an angle of 90º. The most medial aspect of the navicular was marked. The height of the navicular was measured using a ruler. Thereafter, the participant was asked to stand on one leg by flexing the contralateral knee. The single-limb stance position was used because recent work by McPoil and Cornwall has shown that measurements taken from this position more accurately represent the position of the foot during the mid-stance phase of walking [15]. Again, the height of the navicular was measured using a ruler. The difference between the heights of the navicular in seated position vs. standing on one leg was recorded as navicular drop. The foot posture index consists of six items used to quantify and classify foot posture [16, 17]. These are (i) palpation of the head of the talus; (ii) curvatures above and below the lateral malleolus; (iii) position of the calcaneus in the frontal plane; (iv) prominence of the malleolus; (v) congruence of the medial longitudinal arch; and (vi) abduction/adduction of the forefoot. Each item was rated on a visual analog scale ranging from –2 to + 2, resulting in a total score of –12 to + 12. Negative values indicate a supinated foot posture, and positive values indicate a pronated foot posture. Of note, values of 10–12 in the foot posture index were classified as over-pronated feet [16, 17]. A detailed description of the foot posture index can be found elsewhere [16, 17]. Table 1 illustrates the group characteristics. The participants were recruited among citizens living in Ardabil city through announcements in the world wide web and social media. Previous studies have shown a strong association between increased sedentary behavior and being overweight in different age groups [18, 19]. For this purpose, we only selected sedentary individuals to be included in this study. We further considered less than 1000 daily steps (recorded using a pedometer App that was installed on the participants’ mobile phone) and no regular physical exercise (questionnaire) defined as less than one weekly exercise session as a sedentary lifestyle [20, 21]. Exclusion criteria for all groups were as follows: weekly practice of 2–3 sessions of physical exercise; history of musculoskeletal surgery at the trunk and/or lower limbs, cardiorespiratory, neuromuscular or orthopedic disorders (except foot pronation for NP and OP participants); and lower limbs length difference larger than 5 mm. All participants were heel strikers as determined by kinetic data. Participants were aged between 18 and 35 years. The research protocol was approved by the ethics committee of the University of Mohaghegh Ardabili, Iran (IR.UMA.REC.1401.095 for females and IR.UMA.REC.093 for males). Prior to the start of the study, all participants provided their written informed consent to participate after benefits and potential risks were explained.

**Table 1:** Anthropometric characteristics of the four experimental groups

	NN (*n* = 22)	NP (*n* = 21)	ON (*n* = 21)	OP (*n* = 20)
Female/male	12/10	12/9	9/12	8/12
Overweight/Obese	0/0	0/0	17/4	15/5
Age (years)	23.2 ± 2.9	23.6 ± 4.2	25.0 ± 4.4*	26.4 ± 5.7*
Body height (cm)	170.4 ± 11.1	171.2 ± 9.3	170.0 ± 10.6	169.1 ± 10.5
Body mass (kg)	62.8 ± 8.5	64.4 ± 9.6	82.4 ± 9.0*	84.0 ± 15.1*
BMI (kg/m2)	21.5 ± 1.4	21.8 ± 1.9	28.7 ± 4.0*	29.1 ± 3.0*
Nav. Drop (cm)	5.8 ± 1.3	**12.0 ± 1.5**^**†**^	6.7 ± 1.5	**12.5 ± 1.8**^**†**^
Lower limbs length difference (mm)	1.2 ± 0.8	1.3 ± 0.8	1.5 ± 1.1	1.0 ± 0.7
Foot posture index	3.8 ± 0.8	**10.8 ± 0.7**^**†**^	3.6 ± 1.0	**10.9 ± 0.9**^**†**^

NN = non-excessive body weight/non-pronated foot; NP = non-excessive body weight/pronated foot; ON = excessive body weight/ non-pronated foot; OP = excessive body weight/pronated foot Nav. Drop = Navicular drop

^*^ denotes significant difference in relation to excessive body weight groups (ON and OP, *p* < 0.0001)

^†^ denotes significant difference in relation to pronated groups (NP and OP, *p* < 0.00001)

### Experimental Protocol

The experimental protocol started with a warm-up consisting of 4-min dynamic stretching and 5-min jogging. Then, participants were familiarized with the 18-m overground walkway with a force plate embedded at its midpoint. The task for the runners was to run through the walkway in ~ 5.6 s (average speed ~ 3.2 m/s, [22] and step with the dominant foot in the middle of the force place [23]. Several familiarization trials were performed for participants to assimilate the required running speed and foot placement on the force plate, while a 10%-time variability was allowed within the trials. Following familiarization, participants performed twenty running trials in which GRFs and moments were recorded. Trials were repeated if the participant did not place the full dominant foot within the limits of the force plate, altered the running pattern to hit the force plate, or if the running speed was above or below 10% of the targeted speed. Participants were allowed to use their preferred running shoes in the study.

### Data Collection and Analysis

**Fig. 1:**
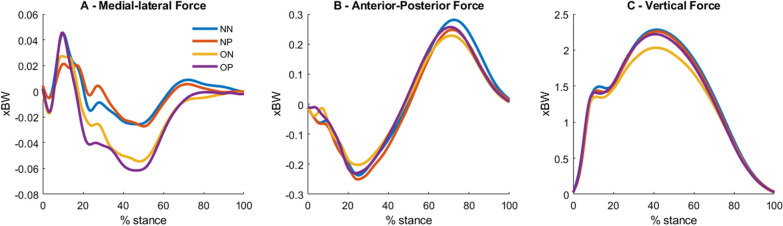
Within-group grand-average ground reaction forces in the medio-lateral **A**, anterior–posterior **B** and vertical directions **C** from the non-excessive body weight/non-pronated foot (NN), non-excessive body weight/pronated foot (NP), overweight/non-pronated foot (ON) and overweight/pronation groups (OP). All force data were normalized to body mass (xBW)

Three-dimensional GRFs and moments were recorded using a force plate (Bertec Corporation, Columbus, 4060–07 Model, OH, United States) sampled at 1000 Hz. Kinetic data were analyzed during the stance phase of running, defined as the interval from ground contact (vertical GRF > 15 N) to toe off (vertical GRF < 15 N). Kinetic data were filtered using a third-order low-pass Butterworth filter with a cutoff frequency of 50 Hz. All GRFs were normalized to the individual’s body mass (xBW). Figure 1 illustrates the grand-averages from the three-dimensional GRFs. Additional file 1: Supplementary Figure 1 illustrates the same data containing the standard deviation patterns for all groups.

The vertical force recorded during running provided the foot contact time, active peak (defined as the highest peak after the impact peak) and the impact peak (defined as the first peak in the vertical force). The time to reach the impact peak was computed from ground contact to the time of impact peak. The vertical impulse was calculated by extracting the area under the vertical force curve. If no impact peak was present, the highest tangential angle within the first 100 ms of stance was used to determine the impact peak [24]. The vertical LR was computed as the average slope from 20 to 80% of the impact peak force [23, 25]. Peak braking and propulsion forces were defined from the lowest and highest force values taken from the anterior–posterior force measurements. Regarding medio-lateral forces, the medio-lateral LR was calculated from the peak medial-to-peak lateral force within the first 20% of the stance phase divided by peak medial-to-peak lateral time. All GRF-related variables were normalized to body mass. Finally, peak negative FM was calculated from the relationship between center of pressure and anterior–posterior/medio-lateral moments as follows [26]:

$$FM={M}_{z}- {F}_{y}\left({\mathrm{CoP}}_{x}\right)+{F}_{x}({\mathrm{CoP}}_{y})$$

where *M*_*z*_ is the vertical moment, *F*_*y*_ and *F*_*z*_ are the anterior/posterior and vertical forces, respectively, and CoP_*z*_ and CoP_*y*_ are the medio-lateral and anterior–posterior center of pressure, respectively.

### Statistical Analyses

The extracted running variables were averaged across 20 trials for each participant. Data are presented as group mean values and standard deviations. We examined and confirmed that the dependent variables (foot contact time, vertical impulse, active peak, time to active peak, impact peak, vertical LR, peak braking and propulsion forces, time to the peak braking and propulsion forces, medio-lateral LR and FM) were normally distributed using the Kolmogorov–Smirnov test. All analyses were performed using custom-made scripts (Matlab R2022a, The MathWorks, Natick, USA). The main effects of body weight (non-excessive body weight, excessive body weight) and foot pronation (non-pronated foot, pronated foot) were computed using a two-way ANOVA for each dependent variable. Effect sizes were estimated using ETA squared (0.01 < ETA ≤ 0.06: small effect size (ES); 0.06 > ETA < 0.14: moderate effect size; ETA ≥ 0.14: large effect size). The significance level was set at *p* < 0.05.

## Results

The anthropometric characteristics of the four experimental groups are presented in Table 1. The non-excessive body weight participants (NN and NP) compared with the excessive body weight groups were chronologically younger (F = 6.6, *p* = 0.01, ES = 0.06), had lower body mass (F = 81.0, *p* = 0.00001, ES = 0.45), and a lower BMI (F = 162.4, *p* < 0.00001, ES = 0.06). In addition, the foot pronated participants (NP and OP) presented greater navicular drop values when compared to non-pronated feet runners (F = 354.5, *p *< 0.00001, ES = 0.77). Test–re-test reliability for running speed data demonstrated ICC > 0.94 for each group and in total.

**Fig. 2:**
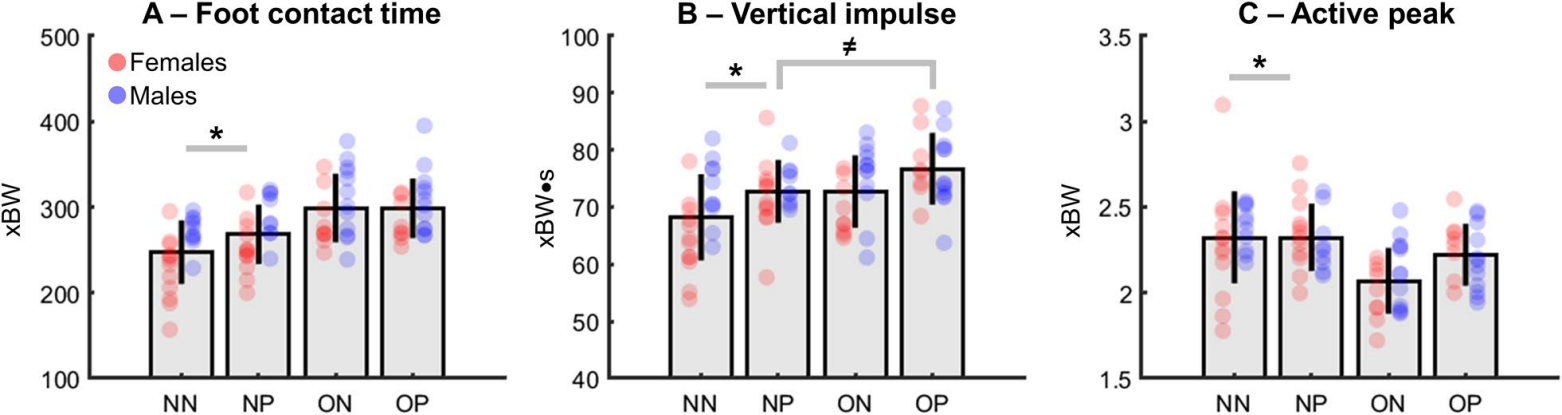
Means ± standard deviations for foot contact times **A**, vertical impulses **B** and active peaks **C** from non-excessive body weight/non-pronated foot (NN), non-excessive body weight/pronated foot (NP), overweight/non-pronated foot (ON) and overweight/pronation (OP) groups. Individual data points are shown from females (*red dots*) and males (*blue dots*). * denotes significant main effect of body weight (*p* < 0.005). ≠ denotes significant main effect of foot pronation (*p* < 0.005)

There were statistically significant main effects of body weight for selected running biomechanical parameters. The excessive body weight groups (ON and OP) presented significantly longer foot contact (~ 13%, *F* = 25.9, *p* < 0.0001, ES = 0.23, Fig. 2A), greater normalized vertical impulse (~ 6%, *F* = 8.9, *p* = 0.003, ES = 0.09, Fig. 1B) and lower normalized active peak (− 9%, F = 14.4, *p* = 0.0002, ES = 0.14, Fig. 2C) when compared to the non-overweight groups (NN and NP). Regarding foot pronation, the pronation groups (NP and OP) presented significantly greater normalized impulses when compared to the non-pronation groups (~ 6%, NN and ON, (*F* = 9.1, *p* = 0.003, ES = 0.09; Fig. 2B). No statistically significant body weight by foot pronation interactions were found for these variables.

The excessive body weight groups showed significantly lower (*F* = 5.9, *p *= 0.01, ES = 0.06) peak braking forces (ON: − 0.23 ± 0.05 xBW; OP: − 0.24 ± 0.05 xBW) compared to the non-overweight groups (NN: -0.25 ± 0.06 xBW; NP: − 0.28 ± 0.07 xBW). With respect to peak propulsion forces, the excessive body weight groups (ON: 0.22 ± 0.04 xBW; OP: 0.25 ± 0.05 xBW) presented significantly lower values (*F* = 9.2, *p* = 0.003, ES = 0.09) compared to the non-overweight groups (NN: 0.30 ± 0.07 xBW; NP: 0.26 ± 0.077 xBW).

**Fig. 3:**
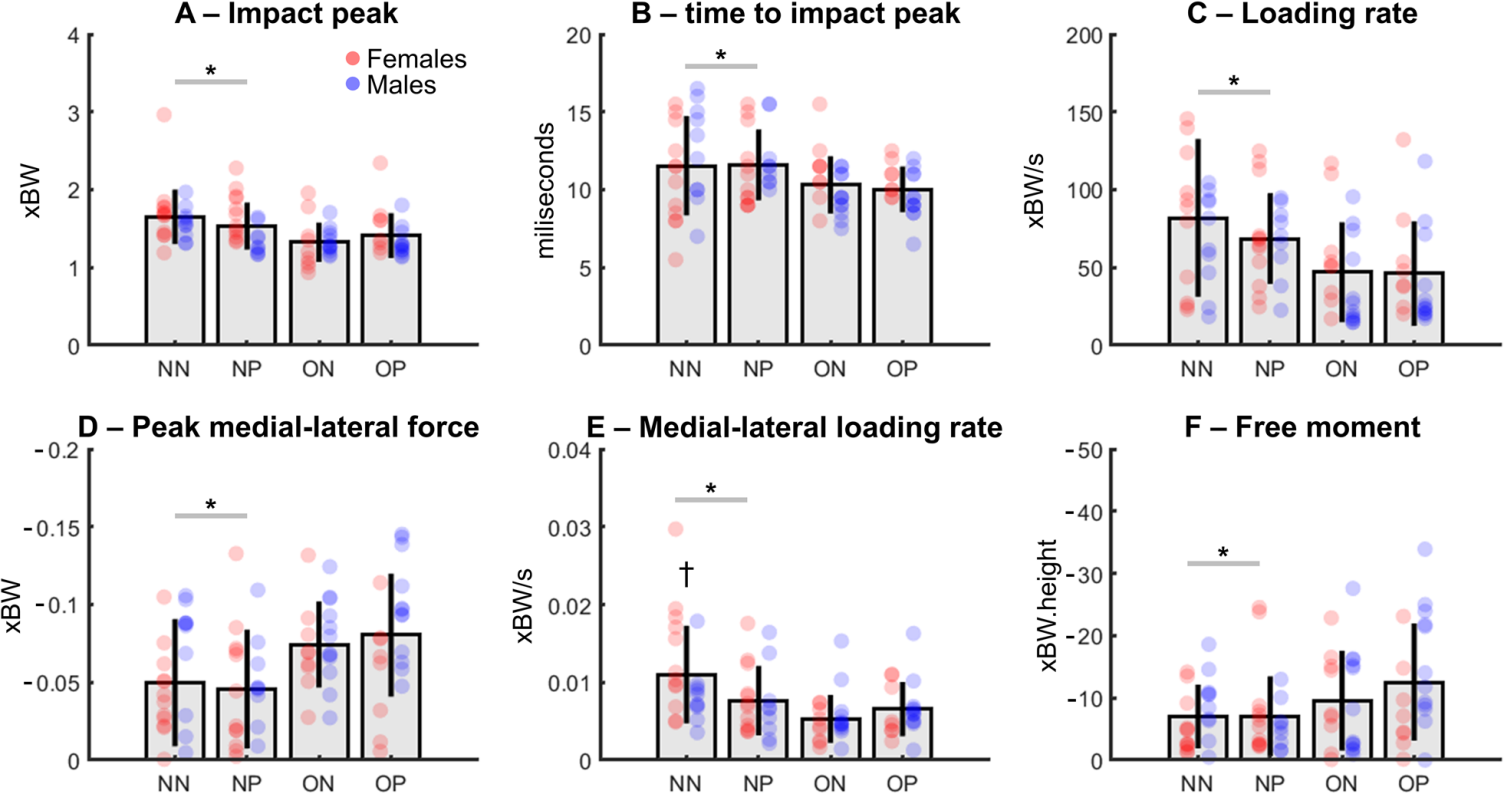
Means ± standard deviations for impact peaks **A**, times to impact peak **B**, vertical loading rates **C**, peak medio-lateral forces **D**, medio-lateral loading rates (**E**) and peak negative free moments** F** from non-excessive body weight/non-pronated foot (NN), non-excessive body weight/pronated foot (NP), overweight/non-pronated foot (ON) and overweight/pronation (OP) groups. Individual data points are shown from females (*red dots*) and males (*blue dots*). * denotes significant main effect of body weight (*p* < 0.01). † denotes significant difference (interaction) in relation to NP, ON and OP groups (*p* < 0.05)

The excessive body weight groups showed significantly lower normalized vertical impact peak forces (− 8%, *F* = 11.69, *p* = 0.001, ES = 0.12, Fig. 3A), shorter times to reach the vertical impact peak (− 6%, *F* = 7.74, *p* = 0.006, ES = 0.08, Fig. 3B) and reduced normalized vertical LR (− 13%, *F* = 12.2, *p* = 0.0007, ES = 0.13, Fig. 3C) when compared to the non-overweight groups (NN and NP). In addition, the excessive body weight groups presented significantly greater peak lateral forces (~ 30%, *F* = 13.4, *p* = 0.0004, ES = 0.14, Fig. 3D), lower normalized medio-lateral LR (− 17%, *F* = 11.7, *p* = 0.0009, ES = 0.12, Fig. 3E), and greater normalized FM (*F* = 6.2, *p* = 0.01, ES = 0.07, Fig. 3F) when compared to the non-overweight groups. Moreover, there were significant body weight by foot pronation interactions for normalized peak of the medio-lateral LR (*F* = 4.5, *p* = 0.03, ES = 0.04, Fig. 3E). Of note, the NN group presented the greatest normalized LR when compared to the other groups (*p* = 0.0001).

## Discussion

This study aimed to investigate the effects of excessive body weight and foot pronation on running kinetics. Our findings revealed that excessive body weight extend the foot-floor interaction and reduces the mechanical responses in the vertical (e.g., reduced impulse, vertical impact peak force) and anterior–posterior directions (e.g., reduced peak braking and propulsion forces). Moreover, individuals with excessive body weight attenuate early vertical impact loading, reducing LR and active peaks at the cost of increasing foot contact time. Our findings are consistent with previous studies reporting up to 20% longer foot contact time during running for young individuals with obesity when compared with young non-excessive body weight individuals [5, 27]. A possible explanation for the group differences in foot contact time may be the poorer balance observed in individuals with excessive body weight, as young overweight and obese individuals have greater postural instability compared with young individuals with non-excessive body weight [28]. Given that there is a lack of research on running biomechanics in adults with excessive body weight, we interpret and discuss our findings with reference to other populations and locomotor modes. Regarding early impact properties, the excessive body weight groups presented lower normalized vertical impact peak forces, shorter time to reach the vertical impact peak and reduced vertical LR when compared to the non-overweight groups (NN and NP). A previous study has demonstrated that obese runners (BMI ≥ 30 kg/m^2^) dampened impact forces and generated lower vertical LR when compared to non-obese runners [6]. Our results corroborate their findings, however, their non-obese group also included individuals with overweight (25 ≤ BMI < 30 kg/m^2^) and there was no control for foot pronation in any group. Therefore, the available studies should be compared with caution. Nonetheless, based on previous literature [6], we speculate that individuals with excessive body weight extend their foot-floor interaction by performing a softer landing, dampening vertical forces and subsequently attenuating the potential damage that early impact forces might cause on their musculoskeletal system. The excessive body weight groups from our study demonstrated greater medio-lateral LR and peak lateral forces (Fig. 3C and D). It has been shown that runners generating higher peak lateral forces are more susceptible to suffer from stress fractures, since high lateral ground reaction forces may increase the stress on bones and joints, leading to stress fractures over time [29]. However, these findings are based on mere statistical correlations that do not allow cause and effect relations. Additionally, there may be other factors, such as deficits in muscle strength and flexibility that contribute to the increased injury risk [30, 31]. In addition to changes in medio-lateral force production, the individuals with excessive body weight examined in this study presented greater peak negative FM (Fig. 3F). FM are caused by frictional forces between the foot and the ground which induce torsional lower limbs stress [32]. Greater peak negative FM may represent greater torsional stress in the supporting limb during running [33], and greater torsional stress may result lower limb injuries such as tibial stress fractures [34]. Also, it has been suggested that FM are relevant to control whole-body angular momentum in the transversal plane during locomotor tasks, being a relevant metric in running mechanics for injury prevention [32, 35]. Therefore, our results contribute to deepening our understanding regarding running-related injuries in individuals with excessive body weight, by demonstrating that this cohort experiences greater torsional forces during running. Moreover, individuals with excessive body weight presented lower peak braking and propulsion forces when compared to the non-excessive body weight groups. Romkes and Schweizer (2015) observed lower hip joint extension during the push-off phase of walking in healthy adults, which may be related to an insufficient recruitment of plantarflexor muscles [36]. Individuals with excessive body weight generate lower plantarflexor and hip extensor moments during the push-off phase of walking which could be a potential gait strategy to maintain upright postural stability [37].

Regarding foot pronation, the pronation groups (NP and OP) presented greater impulses when compared to the non-pronation groups (NN and ON). Notably, the vertical impulse is among others a relevant factor related to running efficiency and performance [38, 39]. Therefore, greater vertical impulses generated by the pronation groups do not necessarily mean greater performance and/or running efficiency.

This study revealed significant body weight by foot pronation interactions for peak propulsion force. The ON group presented the lowest peak propulsion force compared to the other groups. There is evidence in the literature that obese individuals (BMI ≥ 30 kg/m^2^) present lower relative strength and endurance in the ankle plantarflexors and invertor muscles when compared to non-overweight individuals [40]. Given that the ankle plantarflexor muscles mainly contribute to anterior GRF generation during the late stance phase of running [41], the reduced peak propulsion forces of ON observed in this study may be related to plantarflexor muscle weakness. In this study, we did not assess muscle activity or muscle strength which is why this postulated association has to be confirmed in future studies.

Another body weight by foot pronation interaction was found in this study for peak medio-lateral LR. Of note, the NN group presented the greatest LR when compared to the other groups. Previously, it has been shown that muscles account for > 92% of the medio-lateral GRFs during walking [42]. Gravity and velocity-related forces have little contributions to the medio-lateral GRFs during walking [42]. Muscles coordinate medio-lateral acceleration via an interplay between the medial GRFs contributed by the abductors and the lateral GRFs contributed by the knee extensors, plantarflexors, and adductors [42]. Our findings demonstrate that both excessive body weight and pronated feet affect the medio-lateral loading rate while running. Moreover, runners with excessive body weight experience greater lateral and torsional forces during stance. Therefore, our results suggest that the factor body mass has a greater impact on running kinetics than foot pronation, restricting medio-lateral motion and increasing torsional forces during the stance phase. Moreover, our study provides a highly relevant contribution to human movement science by isolating the effects of excessive body weight and foot pronation during running. We were able to demonstrate that factors associated with musculoskeletal disorders (e.g., ankle sprains and stress fractures) appear to be primarily caused by excessive body weight and to a lesser extent by foot pronation.

A study limitation is that we did not collect kinematic data during running which is why we were only able to analyze lower limbs kinetic data during running. Future studies are needed to synchronize kinematic with kinetic data. Although we recorded the average velocity while running on the 18 m walkway and reached ~ 3.2 m/s in average running speed, the instantaneous speed when participants stepped onto the force platform might have varied slightly across trials and participants. It is noteworthy that participants were not intentionally accelerating or decelerating when approaching the force platform, as the device was located in the middle of the walkway. Therefore, the instantaneous speed when reaching the force platform may be slightly different from the intended 3.2 m/s across all trials and participants. The use of timing gates or motion capture is relevant to assure similar running speed in such experiments. In addition, test–re-test reliability for running speed data demonstrated ICCs > 0.94 for each group and in total. Our ICC data indicate that participants in each group had similar average running speed. Because footwear can significantly influence impact loading, the free selection of running shoes by the study participants may impact on the study outcomes. For example, a previous study has demonstrated that the impact vertical force and loading rate increased after running until fatigue with neutral shoes, but not with motion-control shoes [23]. These results suggest that motion-control shoes prevent exacerbated fatigue-related increases in mechanical loading following initial contact in pronated female runners [23]. In another study, it has been reported that compared with conventional running shoes and maximalist shoe conditions, minimalist shoes significantly increased the peak impact acceleration of the distal tibia [43]. Shock attenuation depicted no difference between shoe conditions but was greater in the maximalist shoes compared with the minimalist shoe condition [43]. Moreover, it has been reported that highly cushioned maximalist shoes alter spring-like running mechanics and amplify rather than attenuate impact loading [44]. However, none of our participants used minimalist shoes in our experiment. Moreover, a limitation of this study is that we cannot completely rule out that different shoe brands and cushioning systems may have had an impact on our data. However, it has previously been established that shoe comfort has an effect on running performance [45] as well which is why preferred shoes may also have a positive impact [45]. In an attempt to address this issue, future studies could use similar running shoes to avoid a potential influence of running shoe material on study outcomes. Sedentary behavior was assessed using two different approaches. First, we used a pedometer app (i.e., pedometer, Pacer Health, Inc, LinkedIn) installed on the participants’ mobile phone to test whether they really performed less than 1000 steps per day. Second, we used a questionnaire and kindly asked the study participants the number of exercise hours per week [20, 21]. Future studies should use accelerometer-based physical activity data or a validated questionnaire such as the International Physical Activity questionnaire (IPAC). Finally, our cohort included overweight (25 ≤ BMI < 30) and obese (30 ≤ BMI ≤ 35) individuals. Yet, the number of participants in these subgroups did not allow to additionally analyze overweight versus obese individuals. This should be examined in future studies.

## Conclusions

Our results suggest that excessive body weight has an impact on ground reaction forces during running. We particularly noted an increase in medio-lateral and torsional forces during the stance phase. Individuals with excessive body weight (overweight and obese adults) appear to adapt their running patterns in an effort to attenuate early vertical impact loading.

### Supplementary Information

**Additional file 1. Supplementary Figure 1.** Within-group grand-average and ±1 standard deviation of ground reaction forces in the medial-lateral (**A**), anterior-posterior (**B**) and vertical directions (**C**) from the non-excessive body weight/nonpronated foot (NN), non-excessive body weight/pronated foot (NP), overweight/non-pronated foot (ON) and overweight/pronation groups (OP). All force data were normalized to body mass (xBW).

## Data Availability

The datasets used and/or analyzed during the current study are available from the corresponding author on reasonable request.

## Abbreviations

BMI: Body mass index
NN: Non-excessive body weight and non-pronated foot
NP: Non-excessive body weight and pronated feet
ON: Excessive body weight individuals with non-pronated foot
OP: Overweight/obese individuals with foot pronation
GRFs: Ground reaction forces
LR: Loading rate
FM: Free moments
ES: Effect size
xBW: Body mass
