# Supplementary material for: The Impact of Excessive Body Weight and Foot Pronation on Running Kinetics: A Cross-Sectional Study
Source: Sports Med Open. 2023 Dec 6;9:116. doi: 10.1186/s40798-023-00663-8 (PMC10700293; doi:10.1186/s40798-023-00663-8)
Supplement: Supplementary file 1 — Additional file 1. Supplementary Figure 1. Within-group grand-average and ±1 standard deviation of ground reaction forces in the medial-lateral (A), anterior-posterior (B) and vertical directions (C) from the non-excessive body weight/nonpronated foot (NN), non-excessive body weight/pronated foot (NP), overweight/non-pronated foot (ON) and overweight/pronation groups (OP). All force data were normalized to body mass (xBW). [file 40798_2023_663_MOESM1_ESM.docx]

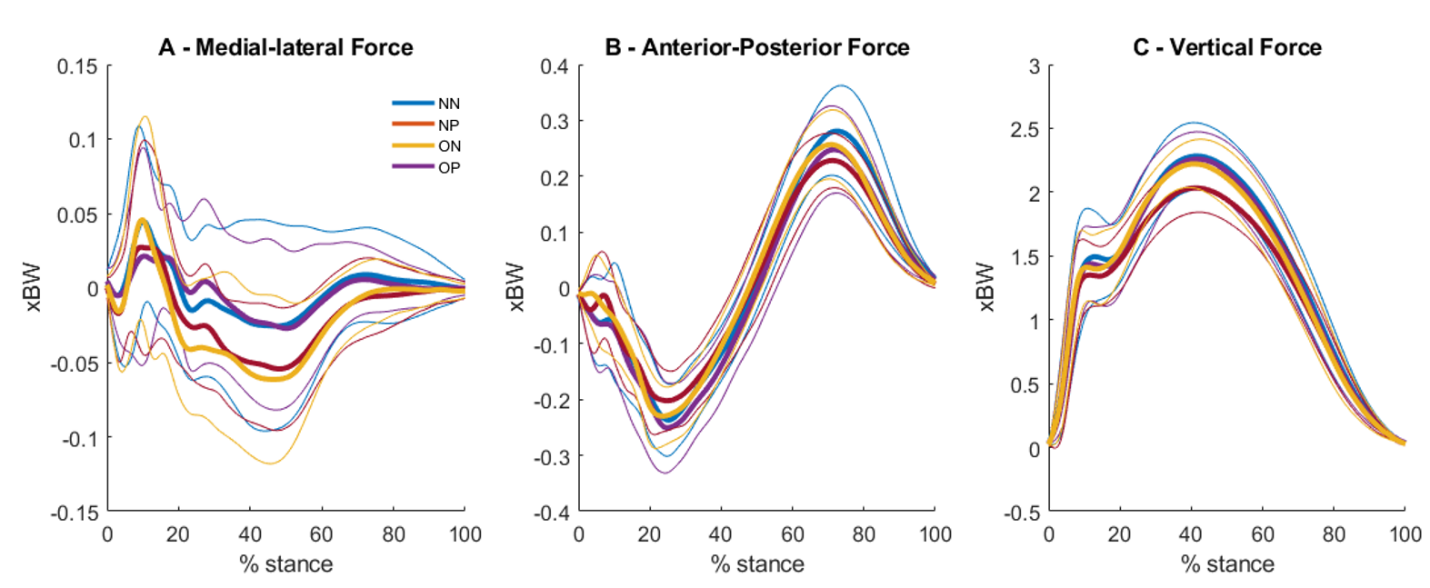


**Supplementary Figure 1**. Within-group grand-average and ±1 standard deviation of ground reaction forces in the medial-lateral (A), anterior-posterior (B) and vertical directions (C) from the non-excessive body weight/non-pronated foot (NN), non-excessive body weight/pronated foot (NP), overweight/non-pronated foot (ON) and overweight/pronation groups (OP). All force data were normalized to body mass (xBW).
